# How Does Symptom Management Affect Quality of Life in Palliative Care Patients? A Descriptive and Correlation Study

**DOI:** 10.1111/scs.70240

**Published:** 2026-04-03

**Authors:** Gülşah Çamcı, Sıdıka Oğuz, Gülsüm Betül Uçar, Pınar Yarman

**Affiliations:** ^1^ Marmara University, Faculty of Health Sciences, Department of Nursing, Department of Internal Medicine Nursing Istanbul Türkiye

**Keywords:** palliative care, patients, quality of life, symptom management

## Abstract

**Aims:**

This study aimed to determine the relationship between symptom management and quality of life in palliative care patients.

**Methods:**

Data was collected using the Patient Information Form, the Edmonton Symptom Assessment Scale (ESAS), and the EQ‐5D Quality of Life Scale. The ESAS rates each symptom from 0 to 10, with scores ≥ 5 indicating moderate‐to‐severe burden. The EQ‐5D index score ranges from −0.59 to 1, with higher scores representing better health‐related quality of life. The EQ‐5D VAS score ranges from 0 to 100.

**Results:**

According to the ESAS, 54.9% of the patients scored ≥ 5 points for pain, 42.5% for fatigue, 34.6% for insomnia, 20.9% for nausea, 44.4% for loss of appetite, 53.6% for shortness of breath, 33.3% for depression, 35.3% for anxiety, 37.9% for feeling unwell, and 26.8% for constipation. The mean EQ‐5D quality of life score was 0.03 ± 0.45, and the EQ‐5D VAS score was 48.10 ± 11.85. The quality‐of‐life sub‐dimension scores showed that 60.1% had extreme problems with mobility, 69.3% in self‐care, 64.7% in usual activities, 21.6% in pain/discomfort, and 15.7% in anxiety/depression. The patient's quality of life decreased as the days of hospitalization increased. A significant negative correlation was observed between quality of life and symptom severity, indicating that quality of life decreased as symptoms worsened. According to the simple regression analyses, the variables pain, fatigue, insomnia, nausea, loss of appetite, depression, anxiety, feeling unwell, constipation, edema in the legs, age, and body mass index were found to be significant negative predictors of quality of life.

**Conclusion:**

Palliative care patients had moderate symptom control and a poor quality of life.

**Practice Implications:**

Pharmacological and non‐pharmacological methods of symptom control in palliative care should be increasingly used. Providing patients with symptom control can improve their quality of life. Further studies on symptom control in palliative care patients are needed.

## 1 Introduction

The World Health Organization (WHO) defines palliative care as the relief of physical, psychosocial, and spiritual symptoms experienced by patients, as well as the support of family and caregivers and the alleviation of suffering [1]. In palliative care, death is deemed a normal process and is neither delayed nor accelerated; the aim is to facilitate the transition from life to death. Palliative care is concerned with the quality of life rather than the duration. In the past, palliative care was an approach for patients in the terminal period, especially cancer patients, when there was no more treatment; today, it is accepted that palliative care should be carried out in addition to therapeutic approaches for life‐threatening diseases [2]. Palliative care is recognized as a human right and is applied to all age groups, although it is generally perceived to concern older patients [3, 4]. Although cancer patients constitute the majority of the patient population, the list of indications is increasing day by day [3].

Each year, an estimated 56.8 million people require palliative care worldwide, including approximately 25.7 million individuals in the final year of life. However, only about 14% of those in need currently receive such services. Among children requiring palliative care, 98% reside in low‐ and middle‐income countries, with nearly half living in Africa. Unnecessarily restrictive regulations governing access to morphine and other essential controlled medications for palliative treatment constitute a major barrier to adequate care. Palliative care is indicated across a broad spectrum of diseases. The majority of adults in need of palliative services are affected by chronic noncommunicable conditions, including cardiovascular diseases (38.5%), cancer (34%), chronic respiratory diseases (10.3%), HIV/AIDS (5.7%), and diabetes (4.6%). In addition, numerous other conditions may necessitate palliative care, such as chronic kidney failure, chronic liver disease, multiple sclerosis, Parkinson's disease, rheumatoid arthritis, various neurological disorders, dementia, congenital anomalies, and drug‐resistant tuberculosis [1].

Importantly, the scope of palliative care has broadened beyond oncology to encompass a wide spectrum of chronic and progressive conditions, including chronic obstructive pulmonary disease, end‐stage renal disease, heart failure, and other advanced non‐malignant illnesses [5]. Patients living with these conditions experience complex and multidimensional symptom burdens comparable to those of patients with advanced cancer. Empirical findings indicate substantial unmet needs in domains such as daily physical care, psychosomatic distress, social isolation, and healthcare system navigation among patients with advanced non‐malignant chronic diseases and their caregivers [6]. Furthermore, comparative analyses demonstrate that although these patients experience symptom burdens similar in magnitude and complexity to those of oncology populations, they are significantly less likely to access palliative care services [7].

Palliative care includes not only physical and psychological symptom control but also other aspects of care, including social and family support, financial, spiritual or religious preferences, goals of care and advanced care planning. Moreover, palliative care shortens hospital and intensive care stays, reduces unnecessary care and hospitalization costs, eases the burden on family members responsible for the patient's care, and increases family satisfaction with care. A life without pain and suffering is essential in palliative care. If patients have good pain and symptom control, their quality of life can be improved. Palliative care should be provided by a multidisciplinary team. If cultural factors affect care, they should be observed, and symptom management should be provided accordingly [8, 9]. The palliative care patient should be approached with a special personal and humanistic attitude beyond daily clinical practices and procedures [2, 9].

Patients in need of palliative care experience many different symptoms intensively. These symptoms are often multidimensional and negatively affect the patient's quality of life and functioning [10]. Patients with advanced cancer experience multiple physical and psychosocial symptoms [11] If patients receive integrated palliative care at an early stage of the disease, symptom management and quality of life have improved [11, 12, 13]. Palliative care services accept patients and provide the necessary care when symptoms cannot be controlled, or home care is no longer possible. When the severity of symptoms such as pain, dyspnea, insomnia, and anxiety decreases, survival and psychological well‐being increase. Failure to control the symptoms that arise due to diseases and treatments may cause patients to discontinue treatment, reduce the treatment dose, or terminate the treatment [14]. Quality of life and symptom control are the primary goals of palliative care. Therefore, quality of life and symptom management are crucial parameters in evaluating palliative care services [15, 16].

Despite the increasing global recognition of palliative care, context‐specific and systematic evidence regarding symptom burden and quality‐of‐life outcomes remains limited in many regions. The generation of empirical evidence in this field is critically important for strengthening evidence‐based clinical decision‐making processes, ensuring the rational and efficient allocation of healthcare resources, and informing the development of palliative care policies.

The comprehensive assessment of symptom burden and quality‐of‐life indicators through the use of valid and reliable measurement instruments will contribute to the enhancement of multidisciplinary care planning, the improvement of clinical practice, and the optimization of palliative care services at both institutional and national levels. This study aimed to determine symptom management and quality of life in palliative care patients.

## 2 Methods

### 2.1 The Purpose of the Research

A descriptive and correlation study was used to determine the relationship between symptom management and quality of life in palliative care patients.

### 2.2 Research Questions

- What are the symptom levels in palliative care patients?
- What is the quality of life of palliative care patients?
- Is there a significant relationship between symptom management and quality of life in palliative care patients?
- What is the effect of selected variables (pain, fatigue, insomnia, nausea, loss of appetite, shortness of breath, depression, anxiety, feeling unwell, constipation, edema in the legs, age and body mass index) on quality of life in palliative care patients?

### 2.3 Setting

The study was conducted from May 1, 2023, to October 31, 2023, in a state's palliative care inpatient clinics and a training and research hospital in Istanbul, Türkiye. There are four palliative care services in the state hospital. Each clinic has 20 beds and can accommodate a total of 80 palliative care patients. There are three palliative care services in the training and research hospital, which accepts a total of 60 patients.

### 2.4 Participants

Inclusion criteria for the study were as follows: consent to participate, no visual or hearing impairment, over 18 years of age, able to answer the questionnaire independently or with the help of the researcher, and being in the palliative care unit for at least 1 week.

Patients were excluded if they had a problem that prevented them from answering the questions (e.g., visual, hearing, speech or comprehension difficulties), wanted to voluntarily withdraw from the study during the study period or had been diagnosed with Alzheimer's disease and dementia.

### 2.5 Sample of the Study

The sample size was calculated using the program G*Power 3.1.9.7 [17]. The sample was calculated for the correlation: bivariate normal model. In the calculation, the sample number was calculated as 84, assuming two tails, an effect size of 0.30 (*r* = 0.30) [18, 19], a margin of error of 5 (*α* = 0.05), a power of 80% (1‐β = 0.80), and an h0 correlation value of 0. However, the study aimed to achieve the largest sample size and reached 159 patients. After six exclusions, the study comprised 153 patients. Three patients were excluded from the study because they did not agree to participate, and three were excluded because they did not speak Turkish. In the post hoc power analysis conducted as a result of the study, the power was determined to be 0.999: Two‐way effect size: 0.414 (correlation between ESAS total score and EQ‐5D index score *r*: 0.414), total sample size 153, and h0 correlation value 0. The sample size was considered sufficient.

### 2.6 Ethical Aspects of the Study

Ethical approval was obtained from the non‐interventional clinical trials ethics committee of Marmara University, Faculty of Health Sciences (29.12.2022/132). Before each data collection interview, the researcher verbally explained the purpose of the study to potential participants. Individuals were then asked to volunteer to participate. Written and verbal consent was obtained for their participation. Participants were informed that they could withdraw from the study at any time. In order to ensure the protection of individual rights during the study, strict adherence was given to the principles outlined in the Declaration of Helsinki on human rights [20]. The data were securely stored and only accessible to authorized researchers. Participants' identities and data were kept confidential and used for research purposes only. All participants were treated equally and fairly without any prejudice or discrimination.

In this study, palliative care patients, who are recognized as a vulnerable population, were systematically evaluated, and ethical safeguards appropriate to their vulnerability were meticulously implemented. In order to protect participants' rights, the processes of informed consent, voluntariness, confidentiality, non‐maleficence, and ethics committee approval were carried out comprehensively. During the informed consent process, participants were provided with clear and comprehensible information regarding the purpose, scope, potential risks, and anticipated benefits of the study. It was explicitly emphasized that participation was entirely voluntary and that participants had the right to withdraw from the study at any stage without providing any justification. Furthermore, ethical sensitivity in this study was regarded not merely as a legal obligation, but as a fundamental component of scientific responsibility and research integrity. Accordingly, preventive and supportive measures were adopted to safeguard the physical and psychosocial well‐being of the participants.

### 2.7 Data Collection Method and Tools

Data collection tools included the Patient Information Form, the Edmonton Symptom Assessment Scale (ESAS), and the EQ‐5D Quality of Life Scale. The researchers collected the data in personal interviews lasting around 25 min in an inpatient palliative care clinic.

#### 2.7.1 Patient Information Form

The Patient Information Form was prepared by reviewing the literature [11, 13, 21] and consists of nine questions about the patient's demographic (gender, smoking and alcohol use status, etc.) and medical characteristics (medications, number of days in hospital, etc.).

#### 2.7.2 Edmonton Symptom Assessment Scale (ESAS)

Bruera et al. [22] developed the Edmonton Symptom Assessment Scale to assess patients' symptoms. The ESAS involves a total of 10 symptoms: fatigue, pain, feeling unwell, nausea, depression, anxiety, sleeplessness, dyspnea, anorexia, and others. Each symptom is scored between 0 and 10. A score of zero indicates no symptoms, and a score of 10 indicates severe symptoms [22]. The validity and reliability of the ESAS in Türkiye were assessed by Yesilbalkan et al. [23]. The Cronbach's *α* value of the scale was 0.77 [23]. In this study, the Cronbach's *α* value of the scale was 0.898.

#### 2.7.3 EQ‐5D Quality of Life Scale

EQ‐5D is a comprehensive health assessment tool used to measure quality of life. It was developed in 1987 by the EuroQol group, the Western European Quality of Life Research Society. It consists of five dimensions: self‐care, movement, pain/discomfort, usual activities, and anxiety/depression. Each dimension is rated on a three‐point Likert scale as having no difficulty, moderate difficulty, and extreme difficulty. Scores between 1 and 3 are obtained from the sub‐dimensions of the scale. A high score indicates a bad situation. A total score ranging from −0.59 to 1 is obtained from the scale. Negative numbers represent circumstances such as unconsciousness, bedridden, etc., while 0 indicates death, and 1 indicates perfect health. In the total scale, higher scores indicate better quality of life. The scale also includes a visual analogue scale (VAS: Visual Analog Scale) in which the daily health status of the individual is evaluated between 0 and 100. The scale can be assessed at any time during the patient's hospitalization and measures their overall quality of life [24]. In Türkiye, Kahyaoğlu Süt and Ünsar (2011) found the scale to be valid and reliable. The Cronbach's *α* value of the scale was 0.860 [25]. In this study, the Cronbach's α value of the scale was 0.795.

### 2.8 Data Evaluation

The data obtained in the study were evaluated by the IBM SPSS v.21 (IBM Corp., Armonk, New York, USA) statistical program. Skewness and kurtosis values were analysed to determine whether the research variables showed normal distribution. The skewness values of the ESAS score were −0.233 to 1.020, and the kurtosis values were −0.591 to 0.1.024. The skewness value of the EQ‐5D quality of life score was 0.635, and the kurtosis value was −0.591. The results of the kurtosis and skewness values of the variables between +1.5 and −1.5 [26], +2.0 and −2.0 [27] are accepted as normal distribution. The variables showed a normal distribution. Parametric methods were used to analyse the data. The relationships between the scale level of the patients and continuous variables were analysed using Pearson correlation analysis. Correlation coefficients (*r*) were evaluated as 0.00–0.10 insignificant; 0.10–0.39 weak; 0.40–0.69 moderate; 0.70–0.89 high; 0.90–1.00 very high [28]. Independent group *t*‐tests, one‐way analysis of variance (ANOVA) and post hoc (Bonferroni) analyses were used to determine the differences between the scale scores according to the descriptive characteristics of the patients. Cohen (*d*) and Eta square (*η* ^2^) coefficients were used to calculate the effect size. The effect size indicates whether the difference between the groups is large enough to be considered significant. Cohen's *d* value is considered 0.2 (small), 0.5 (medium), and 0.8 (big). The eta squared value is considered 0.01 (small), 0.06 (medium), and 0.14 (big) [29]. Simple linear regression and multiple linear regression analysis (enter model) was performed to determine the effects of some variables on quality of life.

## 3 Results

### 3.1 Patients' Descriptive Characteristics

Of the patients, 50.3% were male, 96.7% were married, 40.5% were literate, 72.5% were ≥ 65 years of age, and 49% had balanced income and expenses; 51.6% had quit smoking, 46.4% had never smoked, and 83.7% had never used alcohol; 30.7% had been hospitalized for 22–28 days and 34.6% for ≥ 29 days; 41.2% were hospitalized with a diagnosis of cancer, 28.8% with chronic obstructive pulmonary disease (COPD), 26.7% with cerebrovascular accident (CVA), and 3.3% with heart failure (Table 1).

**TABLE 1:** Distribution of patients according to descriptive characteristics (*N* = 153).

Variables		*n*	%
Gender	Female	76	49.7
	Male	77	50.3
Marital status	Married	148	96.7
	Single	5	3.3
Educational status	Literate	62	40.5
	Primary school‐secondary school	39	25.5
	High school and above	52	34
Age, mean ± SD (min‐max) = 71.019 ± 13.803 (18–101)	≤ 64	42	27.5
	≥ 65	111	72.5
Economical situation	Income expense balanced	75	49
	Income more than expenses	52	34
	Income less than expenses	26	17
Smoking status	Never used	71	46.4
	Quit	79	51.6
	Active	3	2
Alcohol used status	Never used	128	83.7
	Quit	25	16.3
Number of days in hospital	≤ 14 gün	25	16.3
	15–21 days	28	18.4
	22–28 days	47	30.7
	≥ 29 days	53	34.6
Medical diagnosis	Cancer	63	41.2
	COPD	44	28.8
	CVA	41	26.7
	Heart failure	5	3.3

Abbreviations: COPD, chronic obstructive pulmonary disease; CVA, cerebrovascular accident.

### 3.2 Edmonton Symptom Assessment Scale and Quality of Life Scores

According to the ESAS, 54.9% of the patients got ≥ 5 points due to pain, 42.5% due to fatigue, 34.6% due to insomnia, 20.9% due to nausea, 44.4% due to loss of appetite, 53.6% due to shortness of breath, 33.3% due to depression, 35.3% due to anxiety, 37.9% due to feeling unwell, 26.8% due to constipation, and 13.7% due to oedema in the legs. It was also detected that 39.2% of the total ESAS scores were ≥ 5 points. The mean pain score was 4.74 ± 2.10, fatigue 3.71 ± 2.18, insomnia 3.52 ± 1.86, nausea 2.10 ± 2.40, loss of appetite 3.42 ± 2.58, shortness of breath 4.57 ± 1.94, depression 3.51 ± 1.75, anxiety 3.51 ± 1.84, feeling unwell 3.61 ± 1.89, constipation 2.25 ± 2.74, oedema in the legs 1.28 ± 2.12, and total ESAS 3.29 ± 1.49 (Table 2).

**TABLE 2:** Distribution of patients' ESAS ve EQ‐5D quality of life scale scores (*N* = 153).

	Score	*n*	%	Mean ± SD	Min	Max
Pain	≤ 4	69	45.1	4.74 ± 2.10	0	9
	≥ 5	84	54.9			
Fatigue	≤ 4	88	57.5	3.71 ± 2.18	0	8
	≥ 5	65	42.5			
Insomnia	≤ 4	100	65.4	3.52 ± 1.86	0	10
	≥ 5	53	34.6			
Nasuea	≤ 4	121	79.1	2.10 ± 2.40	0	8
	≥ 5	32	20.9			
Loss of appetite	≤ 4	85	55.6	3.42 ± 2.58	0	8
	≥ 5	68	44.4			
Shortness of breath	≤ 4	71	46.4	4.57 ± 1.94	0	8
	≥ 5	82	53.6			
Depression	≤ 4	102	66.7	3.51 ± 1.75	0	7
	≥ 5	51	33.3			
Anxiety	≤ 4	99	64.7	3.51 ± 1.84	0	7
	≥ 5	54	35.3			
Feeling unwell	≤ 4	95	62.1	3.61 ± 1.89	0	8
	≥ 5	58	37.9			
Constipation	≤ 4	112	73.2	2.25 ± 2.74	0	9
	≥ 5	41	26.8			
Edema in the legs	≤ 4	132	86.3	1.28 ± 2.12	0	7
	≥ 5	21	13.7			
Total ESAS	≤ 4	93	60.8	3.29 ± 1.49	0.82	6.36
	≥ 5	60	39.2			

EQ‐5D quality of life scale scores of patients						
Sub‐dimensions	No problem		Some problems		Extreme problem	
	*n*	%	*n*	%	*n*	%
Mobility	21	13.7	40	26.2	92	60.1
Self‐Care	23	15.0	24	15.7	106	69.3
Usual activities	14	9.2	40	26.1	99	64.7
Pain/discomfort	21	13.7	99	64.7	33	21.6
Anxiety/depression	21	13.7	108	70.6	24	15.7
	**Mean**	**SD**	**Min**	**Max**	**Scale min–max**	
EQ‐5D index score	0.03	0.45	−0.59	0.92	−0.59‐1	
EQ‐5D VAS score	48.10	11.85	20	80	0–100	

Abbreviation: VAS, Visual Analog Scale.

It was concluded that 60.1% of the patients had extreme problems with movement, 69.3% in self‐care, 64.7% in usual activities, 21.6% in pain/discomfort, and 15.7% in anxiety/depression. The mean EQ‐5D index score was 0.03 ± 0.45, and the EQ‐5D VAS score was 48.10 ± 11.85 (Table 2).

### 3.3 Comparison of Patients' Descriptive Characteristics With Quality of Life Scores

There was a difference in EQ‐5D VAS score according to economic status (*F* (2, 150) = 3.256, *p* = 0.041, *Ƞ* ^2^ = 0.042). The post hoc analysis found that the EQ‐5D VAS score of patients with balanced income was higher than that of those with less income. This finding supports the view that economic adequacy may have an effect on health‐related quality of life.

Patients aged 65 years and older had a lower EQ‐5D index score (*t* = 2.669; *p* = 0.008; *d* = 0.449; *η* ^2^ = 0.045). This finding indicates that patients in the older age group have significantly lower quality‐of‐life levels compared to younger patients.

There was a significant difference between the number of days of hospitalization and EQ‐5D index (*F* (3, 149) = 31.207, *p* = < 0.001, *Ƞ* ^2^ = 0.386) and EQ‐5D VAS score (*F* (3, 149) = 27.861, *p* = < 0.001, *Ƞ* ^2^ = 0.359). In post hoc analysis, the EQ‐5D index and EQ‐5D VAS scores of patients with less than 14 days of hospitalization were higher than those of the other groups. This finding suggests that a shorter length of hospital stay may be associated with better perceived health status and quality of life. Conversely, prolonged hospitalization may indicate greater disease severity, increased symptom burden, and functional decline, thereby contributing to a reduction in quality of life (Table 3).

**TABLE 3:** Comparison of descriptive characteristics of patients with EQ‐5D index and EQ‐5D‐VAS score.

		EQ‐5D index score	EQ‐5D VAS score
		Mean ± SD	Mean ± SD
Gender	Female	0.01 ± 0.45	47.89 ± 11.11
	Male	0.04 ± 0.46	48.31 ± 12.61
	Statistics	*t* = −0.344 *p* = 0.732	*t* = −0.217, *p* = 0.151
Economical situation	Income expense balanced	0.058 ± 0.51	50.13 ± 13.10 (1)
	Income more than expenses	0.04 ± 0.41	47.50 ± 10.64 (2)
	Income less than expenses	−0.08 ± 0.36	43.46 ± 8.92 (3)
	Statistics	*F* = 0.953, *p* = 0.388	*F* = 3.256, ***p* = 0.041**, **1 > 3**
Age	≤ 64	0.18 ± 0.51	50.71 ± 13.14
	≥ 65	−0.03 ± 0.42	47.12 ± 13.14
	Statistics	*t* = 2.669, ***p* = 0.008**	*t* = 1.686, *p* = 0.094
Number of days in hospital	≤ 14 gün	0.65 ± 0.42 (1)	63.60 ± 12.54 (1)
	15–21 days	−0.015 ± 0.38 (2)	45.71 ± 10.69 (2)
	22–28 days	−0.05 ± 0.36 (3)	47.23 ± 9.48 (3)
	≥ 29 days	−0.17 ± 0.30 (4)	42.83 ± 7.17 (4)
	Statistics	*F* = 31.207, ***p* < 0.001**, 1 > 2, 3, 4	*F* = 27.861, ***p* < 0.001**, 1 > 2, 3, 4

*Note:* Bold formatting was used to highlight statistically significant results.

Abbreviations: *F*, one way ANOVA *p* < 0.05; *t*, student *t*‐test.

### 3.4 Correlation Between ESAS, EQ‐5D Index, EQ‐5D VAS, Age and Hospitalization Day

There was no correlation between age and Edmonton Symptom Assessment Scores (*p* > 0.05). There was a weak negative correlation between age and EQ‐5D index (*r* = −0.298, *p* < 0.001). There was a very weak negative correlation between age and EQ‐5D VAS score (*r* = −0.212, *p* < 0.001). The number of days of hospitalization was correlated with pain (*r* = 0.354, *p* < 0.001), fatigue (*r* = 0.247, *p* = 0.002), nausea (*r* = 0.286, *p* < 0.001), loss of appetite (*r* = 0.306, *p* < 0.001), depression (*r* = 0.336, *p* < 0.001), anxiety (*r* = 0.401, *p* < 0.001), feeling unwell (*r* = 0.347, *p* < 0.001) and ESAS total (*r* = 0.345, *p* < 0.001). A moderate negative correlation was found between the EQ‐5D index (*r* = −0.588, *p* < 0.001) and the EQ‐5D VAS score (*r* = −0.553, *p* < 0.001) and the number of days hospitalized. The EQ‐5D index score was correlated with pain (*r* = −0.371, *p* < 0.001), fatigue (*r* = −0.349, *p* < 0.001), depression (*r* = −0.397, *p* < 0.001), anxiety (*r* = −0.452, *p* < 0.001), feeling unwell (*r* = −. 469, *p* < 0.001), constipation (*r* = −0.346, *p* < 0.001) and ESAS total (*r* = −0.414, *p* < 0.001). There was a moderate negative relationship between EQ‐5D VAS score and pain (*r* = −0.526, *p* < 0.001) and feeling unwell (*r* = −0.543, *p* < 0.001). EQ‐5D VAS score was correlated with fatigue (*r* = −0.466, *p* < 0.001), insomnia (*r* = −0.294, *p* < 0.001), loss of appetite (*r* = −0.366, *p* < 0.001), depression (*r* = −0.451, *p* < 0.001), anxiety (*r* = −0.452, *p* < 0.001), constipation (*r* = −0.264, *p* = 0.001) and ESAS total (*r* = −0.482, *p* < 0.001). There was a high positive correlation between the EQ‐5D VAS score and the EQ‐5D index (*r* = 0.781, *p* < 0.001) (Table 4).

**TABLE 4:** Correlation between ESAS, EQ‐5D index, EQ‐5D‐VAS, age and hospitalization day.

		Age	Hospitalization day	EQ‐5D index score	EQ‐5D VAS score
Pain	*r*	−0.001	0.354**	−0.371**	−0.526**
	*p*	0.991	**< 0.001**	**< 0.001**	**< 0.001**
Fatigue	*r*	−0.014	0.247**	−0.349**	−0.466**
	*p*	0.863	0.**002**	**< 0.001**	**< 0.001**
Insomnia	*r*	−0.101	0.108	−0.198*	−0.294**
	*p*	0.212	0.185	0.**014**	**< 0.001**
Nasuea	*r*	−0.062	0.286**	−0.167*	−0.240**
	*p*	0.450	**< 0.001**	0.**039**	0.**003**
Loss of appetite	*r*	0.003	0.306**	−0.244**	−0.366**
	*p*	0.966	**< 0.001**	0.**002**	**< 0.001**
Shortness of breath	*r*	−0.046	−0.123	0.145	0.073
	*p*	0.572	0.130	0.073	0.368
Depression	*r*	0.055	0.336**	−0.397**	−0.451**
	*p*	0.503	**< 0.001**	**< 0.001**	**< 0.001**
Anxiety	*r*	0.093	0.401**	−0.452**	−0.452**
	*p*	0.253	**< 0.001**	**< 0.001**	**< 0.001**
Feeling unwell	*r*	−0.010	0.347**	−0.469**	−0.543**
	*p*	0.900	**< 0.001**	**< 0.001**	**< 0.001**
Constipation	*r*	0.071	0.199*	−0.346**	−0.264**
	*p*	0.383	0.**014**	**< 0.001**	0.**001**
Edema in the legs	*r*	0.074	0.204*	−0.375**	−0.245**
	*p*	0.361	**0.012**	**< 0.001**	**0.002**
Total ESAS	*r*	0.009	0.345**	−0.414**	−0.482**
	*p*	0.910	**< 0.001**	**< 0.001**	**< 0.001**
EQ‐5D index score	*r*	−0.298**	−0.588**	1	0.781**
	*p*	**< 0.001**	**< 0.001**		**< 0.001**
EQ‐5D VAS score	*r*	−0.212**	−0.553**	0.781**	1
	*p*	0.**009**	**< 0.001**	**< 0.001**	

*Note:* Bold formatting was used to highlight statistically significant results.

Abbreviations: ESAS, Edmonton Symptom Assessment Scale; *r*, Pearson correlation.

* < 0.05.

** < 0.01.

### 3.5 Regression Analysis of Selected Variables on Quality of Life

According to the simple regression analyses, the variables pain (*F* (1, 151) = 24.11, *p* < 0.001, *R* ^2^ = 0.14), fatigue (*F* (1, 151) = 20.93, *p* < 0.001, *R* ^2^ = 0.12), insomnia (*F* (1, 151) = 6.78, *p* = 0.010, *R* ^2^ = 0.04), nausea (*F* (1, 151) = 4.34, *p* = 0.039, *R* = 0.03), loss of appetite (*F* (1, 151) = 9.56, *p* = 0.002, *R* ^2^ = 0.06), depression (*F* (1, 151) = 28.32, *p* < 0.001, *R* ^2^ = 0.16), anxiety (*F* (1, 151) = 38.87, *p* < 0.001, *R* ^2^ = 0.20), feeling unwell (*F* (1, 151) = 42.48, *p* < 0.001, *R* ^2^ = 0.22), constipation (*F* (1, 151) = 20.47, *p* < 0.001, *R* ^2^ = 0.12), edema in the legs (*F* (1, 151) = 24.78, *p* < 0.001, *R* ^2^ = 0.14), age (*F* (1, 151) = 14.72, *p* < 0.001, *R* ^2^ = 0.09), and body mass index (*F* (1, 151) = 22.23, *p* < 0.001, *R* ^2^ = 0.13) were found to be significant negative predictors of quality of life. An increase in the scores of these variables was associated with a decrease in quality of life. Among them, feeling unwell emerged as the strongest individual predictor, explaining 22% of the variance in quality of life scores (*R* ^2^ = 0.22) (Table 5).

**TABLE 5:** Effect of the selected variables on quality of life.

Simple regression							Multiple regression				
Variable	*B*	SE	*β*	*t*	*p*	*R* ^2^	*B*	SE	*β*	*t*	*p*
Pain	−0.08	0.02	−0.37	−4.91	**< 0.001**	0.14	−0.005	0.03	−0.02	−0.20	0.838
Faigue	−0.07	0.02	−0.35	−4.58	**< 0.001**	0.12	−0.02	0.02	−0.07	−0.66	0.509
Insomnia	−0.05	0.02	−0.21	−2.60	0.**010**	04	−0.01	0.02	−0.06	−0.74	0.458
Nasuea	−0.03	0.02	−0.17	−2.08	0.**039**	0.03	0.02	0.02	0.13	1.47	0.144
Loss of appetite	−0.04	0.01	−0.24	−3.09	0.**002**	0.06	0.03	0.02	0.20	1.88	0.062
Shortness of breath	0.03	0.02	0.15	1.80	0.073	0.02	0.04	0.02	0.19	2.76	0.**007**
Depression	−0.10	0.02	−0.40	−5.32	**< 0.001**	0.16	0.007	0.03	0.03	0.24	0.809
Anxiety	−0.11	0.02	−0.45	−6.23	**< 0.001**	0.20	−0.05	0.03	−0.18	−1.62	0.108
Felling unwell	−0.11	0.02	−0.47	−6.52	**< 0.001**	0.22	−0.09	0.03	−0.37	−2.94	0.**004**
Constipation	−0.06	0.01	−0.35	−4.52	**< 0.001**	0.12	−0.004	0.01	−0.02	−0.27	0.788
Edeme in the legs	−0.08	0.02	−0.38	−4.98	**< 0.001**	0.14	−0.04	0.02	−0.20	−2.73	0.**007**
Age	−0.010	0.003	−0.30	−3.84	**< 0.001**	0.09	−0.007	0.002	−0.20	−3.10	0.**002**
Body mass index	0.06	0.01	0.36	4.71	**< 0.001**	0.13	0.04	0.01	0.24	3.44	**< 0.001**
							*F* (13, 139) = 9.74, *p* < 0.001, *R* ^2^ = 0.48				

*Note:* Dependent variable: EQ‐5D quality of life scale. Bold formatting was used to highlight statistically significant results.

Abbreviations: *β*, standardized regression coefficient; *B*, unstandardized coefficient; SE, standard error.

In the multiple regression analysis, feeling unwell, edema in the legs, shortness of breath, age, and body mass index were identified as significant predictors of quality of life. Together, the variables included in the model accounted for 48% of the total variance in the dependent variable (*F* (13, 139) = 9.74, *p* < 0.001, *R* ^2^ = 0.48) (Table 5).

## 4 Discussion

According to the ESAS, almost 50% of the patients in this study experienced pain, exhaustion, insomnia, lack of appetite, shortness of breath, depression, anxiety, and feeling unwell (Table 2). In a study conducted on cancer patients receiving palliative care, the highest mean score among symptoms was pain, followed by fatigue, loss of appetite, insomnia, constipation, shortness of breath, and nausea and vomiting [30]. Another study revealed that the most common symptoms included loss of appetite, weight loss, and pain. The least reported symptoms were dizziness, gas, and itching [31]. In the study of Uysal et al., symptom levels were examined at the time of admission and on the seventh day in patients admitted to the palliative care service. It was found that the mean values of pain, insomnia, anorexia, and well‐being symptoms decreased significantly on the third and seventh days after admission compared to the time of admission. Moreover, no statistically significant difference was found between the symptoms of nausea, anxiety, shortness of breath, and constipation [14]. An eight‐month prospective study of 1789 cancer patients from 12 different countries undergoing palliative care revealed that the quality of life, functional and various symptoms except nausea and vomiting remained generally stable over time. In their retrospective analysis, patients' quality of life and physical functioning scores were significantly lower 0–2 months before death than 3–5 months before death. Emotional functioning was unchanged at baseline but decreased in the last few months. Pain, fatigue, and loss of appetite showed a steady increase in intensity towards death [32]. In the study conducted by Grochowicka et al. among palliative care patients, it was found that oncology patients reported higher levels of pain, whereas non‐oncological patients experienced dyspnea more frequently [33]. The prevalence of symptoms in this study is similar to the studies above. Pain was the worst and most common symptom reported. The absence of pain is the most important factor in palliative care. Symptom management in palliative care should be improved, and symptom assessment is important in the evaluation of the services provided. In this study, all patients hospitalized in the palliative care unit were included. In contrast, a substantial proportion of the existing literature has predominantly focused on oncology patients. The inclusion of diverse diagnostic groups in our study demonstrates the presence of a broad and heterogeneous spectrum of symptoms among palliative care patients. Therefore, our findings should be interpreted and generalized with careful consideration of the diversity inherent in the palliative care population.

This study found patients' quality of life to be poor. In the quality‐of‐life sub‐dimensions, 60.1% of the patients had serious problems with movement, 69.3% with self‐care, 64.7% with usual activities, 21.6% with pain/discomfort and 15.7% with anxiety/depression (Table 2). The variables pain, fatigue, insomnia, nausea, loss of appetite, depression, anxiety, feeling unwell, constipation, edema in the legs, age, and body mass index were found to be significant negative predictors of quality of life (Table 5). The study conducted in the last 2 weeks of end‐of‐life care found that patients' quality of life worsened towards death. Physical symptoms and meaning of life were found to be the worst symptoms [34]. A study of cancer patients receiving palliative care in Saudi Arabia found that quality of life had a weak relationship with physical function and satisfaction with the care received, while emotional function and general health status had a moderate relationship with general satisfaction [35]. A study conducted in India observed that more than 50% of patients with advanced cancer had difficulty in daily life, while the majority of patients (77%) had difficulty in doing heavy work [36]. In a meta‐analysis study conducted on palliative care patients with diseases other than cancer, the symptom burden was found to be lower than that of patients receiving routine treatment. No significant difference was found in quality of life [37]. In the study conducted by Wang and Ding among patients with advanced‐stage cancer, it was determined that those who received integrated palliative care had significantly higher quality‐of‐life levels compared with the control group [38]. In a study conducted among patients with advanced neurological disorders, anxiety and depression were found to mediate the relationship between palliative care needs and quality of life [39]. In a study conducted among patients with end‐stage liver disease, the integration of palliative care was found to reduce hospitalizations and improve quality of life [40]. In their study, Jones et al. found poor symptom management and quality of life [41]. In several other studies, patients' quality of life was also poor [42, 43, 44]. The above studies found similar results to this one. The investigations identified a significant decline in physical movement and performance of everyday tasks, both of which are key components of quality of life. Palliative care patients often have a poor quality of life, and one reason for this is poor symptom control [31, 45]. Early‐initiated, holistic, and patient‐centered palliative care provides effective symptom management in cancer and other advanced‐stage diseases. This approach makes a significant contribution to the improvement of patients' quality of life.

### 4.1 Limitations

The main limitation of this study is that a substantial proportion of the patients in the study setting had chronic diseases other than cancer; this makes it difficult to attribute symptom changes and quality of life problems solely to a single disease group. The presence of comorbid conditions may be considered a potential confounding factor that could have influenced the results. Therefore, caution should be exercised when generalizing the findings to a specific patient population.

Studies on symptom management and quality of life in palliative care are insufficient. Symptoms and quality of life worsen as death approaches. This study is significant because the patients who participated were receiving end‐of‐life care. Therefore, this study will contribute to the literature.

## 5 Conclusion

Patients receiving palliative treatment exhibited a moderate level of symptom management and a low quality of life. Symptom management is important for palliative care patients. However, in this study, the observed symptom management was inadequate. In addition, the quality of life was poor. Therefore, pharmacological and non‐pharmacological methods for symptom control in palliative care should be increased. If patients can achieve better symptom control, their quality of life can improve. The results of this study are important for all health professionals to improve the quality of palliative care.

A multidisciplinary and patient‐centred approach should be taken to improve symptom control in palliative care. Opioids, non‐opioid analgesics, and adjuvant medications should be used correctly using the WHO pain grading system. Non‐pharmacological methods (such as physiotherapy, acupuncture, massage, and relaxation techniques) can be used supportively. Dyspnea can be treated with oxygen therapy, opioids, and relaxation techniques. The patient's comfort can be increased by supporting breathing and changing positions. Antiemetic medication (e.g., metoclopramide and ondansetron) can be used for nausea and vomiting. Adequate fluid intake, fibre, and laxatives should be recommended for constipation. Pharmacological and psychotherapeutic approaches can treat depression, anxiety, and sleep disorders. Social support groups, moral support, and psychosocial services should be offered. Energy conservation strategies (rest and adequate exercise) should be encouraged for fatigue. Underlying causes such as anaemia, nutritional deficiencies, and depression should be investigated. These approaches are critical to improving patients' quality of life and controlling their symptoms. There should be effective communication between the patient, family, and medical staff.

## Author Contributions

Gülşah Çamcı, Sıdıka Oğuz, Gülsüm Betül Uçar and Pınar Yarman: writing – review and editing, writing – original draft, methodology, resources and methodology, formal analysis, conceptualization, supervision and resources.

## Funding

The authors have nothing to report.

## Ethics Statement

Ethical approval for the study was obtained from Marmara University, Faculty of Health Sciences, Non‐Invasive Clinical Research Ethics Committee (29.12.2022/132).

## Conflicts of Interest

The authors declare no conflicts of interest.

## Data Availability

The data that support the findings of this study are available from the corresponding author upon reasonable request.
